# Endosome and INPP4B

**DOI:** 10.18632/oncotarget.6663

**Published:** 2015-12-18

**Authors:** Chen Li Chew, Ming Chen, Pier Paolo Pandolfi

**Affiliations:** Cancer Research Institute, Beth Israel Deaconess Cancer Center, Department of Medicine and Pathology, Beth Israel Deaconess Medical Center, Harvard Medical School, Boston, MA, USA

**Keywords:** cancer, metastasis, genetics, INPP4B, endosome, PI3K/AKT, thyroid

**Figure 1:**
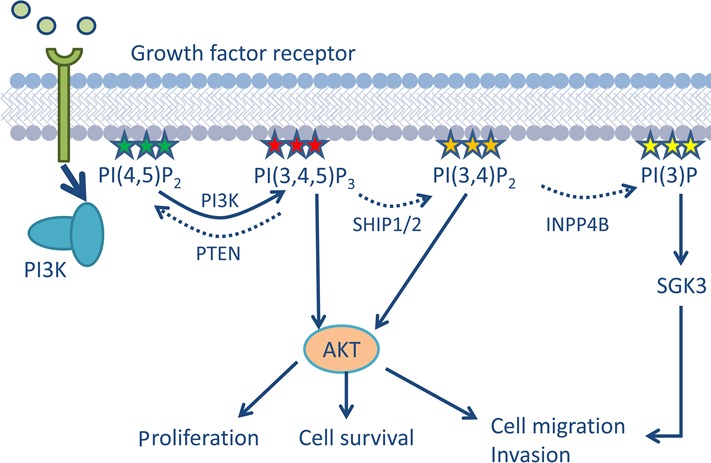
Regulation of PI3K/AKT signaling through phosphatases Growth factor signaling results in the activation of PI3K, which produces PI(3,4,5)P_3_. PI(3,4,5)P_3_ can subsequently be dephosphorylated by SHIP1/2 to PI(3,4)P_2_. Both PI(3,4,5)P_3_ and PI(3,4)P_2_ can activate AKT, leading to cell proliferation, survival, migration and invasion. PTEN and INPP4B dephosphorylate PI(3,4,5)P_3_ and PI(3,4)P_2_ respectively, acting as tumor suppressors by antagonizing AKT signaling. Under certain circumstances, INPP4B can act as an oncogene – PI(3)P, the product of INPP4B action, can activate SGK3, which activates pathways in proliferation, cell survival, migration and invasion. *Figure adapted from Gewinner C et al. Cancer Cell. 2009*.

Phosphoinositide 3-kinase (PI3K) and AKT define a signaling network that regulates important biological processes such as cell cycle, survival, metabolism and motility, all of which are disrupted in cancer (Figure 1) [1]. Hyperactivation of PI3K leads to increased production of phosphoinositide (PI) species such as PI(3,4,5)P_3_ and PI(3,4)P_2_, thereby increasing membrane recruitment and activation of AKT, a key mediator of the oncogenic effects of enhanced PI3K signaling (Figure 1) [1]. Lipid phosphatases, like PTEN, control the levels of PI(3,4,5)P_3_ to antagonize oncogenic PI3K/AKT signaling. INPP4B (inositol polyphosphate 4-phosphatase type II) has recently emerged as an important player in the regulation of PI3K-AKT signaling. INPP4B was initially identified as a potential tumor suppressor in a shRNA-mediated genetic screen performed in HMEC cells, where knockdown of INPP4B resulted in anchorage independent growth [2]. INPP4B preferentially hydrolyzes PI(3,4)P_2_ to PI(3)P [3] and, because the direct interaction of PI(3,4)P_2_ with the pleckstrin homology domain of AKT is required for membrane recruitment and complete activation of AKT [1], INPP4B, like PTEN, should act as a tumor suppressor. The tumor suppressive role of INPP4B has been subsequently confirmed in two independent studies *in vitro*, where knockdown of *INPP4B* in basal-like breast cancer cell lines was found to increase cell proliferation, anchorage-independent growth and migration [3, 4].

Since the tumor suppressive role of INPP4B *in vivo* had not been addressed previously, we sought to further investigate 1) the tumor suppressive function of INPP4B both *in vivo* and *in vitro* with knockout mouse models, and 2) if *Inpp4b* loss cooperates with *Pten* heterozygosity in tumor progression. Our *in vivo* study showed that INPP4B is not solely epistatic to PTEN, since crossing *Inpp4b*−/− mice with *Pten*+/− mice did not accelerate the entire tumor spectrum of *Pten*+/− mice. Critically, we observed a specific acceleration of thyroid adenomas to metastatic follicular thyroid cancer (FTC), which resulted in the early mortality that occurred in the *Pten*+/−*Inpp4b*−/− mice [5]. Further studies using conditional *Pten* and *Inpp4b* knockout mice will be needed to determine the potential cooperative effect between PTEN and INPP4B in controlling tumorigenesis and metastasis in other tissues.

The cooperative roles of PTEN and INPP4B in suppressing tumor growth and metastatic FTC are evident in both our mouse model and in FTC patients where these two tumor suppressors are found concomitantly lost [5, 6]. However, the specificity of such a phenotype suggests that while increased Akt activation plays a role in follicular-like thyroid tumorigenesis, it was insufficient in mediating progression and metastases, It also suggests that the tumor suppressive function of INPP4B was therefore extended beyond its role in suppressing the overall level of PI3K-AKT pathway activation. In this respect, a striking difference between PTEN and INPP4B emerged from our cellular fractionation experiments in their differential localization at the early endosome where INPP4B, but not PTEN, could regulate AKT2 signaling in a localized and specialized fashion [4].

Three highly homologous isoforms of AKT have been identified [1], however, despite their structural similarities, there is increasing evidence that these AKT isoforms are not functionally equivalent, rather, they promote distinct signaling outputs. Of note, AKT1 suppresses, while AKT2 promotes invasion, migration and epithelial-mesenchymal transition [1]. However, the mechanisms underlying specific AKT isoform activation and the identity of isoform specific substrates remain poorly understood. Through cellular fractionation experiments, we have provided evidence for the selective regulation of AKT2 by INPP4B through its localization at early endocytic membranes.

Interestingly, PTEN has also recently been reported to be present on PI(3)P vesicles [7]. Although the exact identity and the cell type specificity of these vesicles remains to be determined, they could represent other endocytic signaling intermediates. The presence of tumor suppressive phosphatases on these membranes underscores the importance of regulating endocytic signaling intermediate, and provides an important link between endocytosis and cancer.

Surprisingly, recent studies have also indicated the oncogenic function of INPP4B in leukemia, breast, and colon cancer. This could be largely attributed to the product of INPP4B phosphatase activity (PI(3)P), which can drive AKT-independent and SGK3-mediated tumorigenesis [8], [*Blood* PMID: 25736236, *Oncogene* PMID: 26411369]. Similar to TGFβ and Notch signaling pathways, the dual activity exhibited by INPP4B in cancer regulation highlight the complexity of lipid signaling and endocytosis in cancer. The paradoxical nature of INPP4B's function could also potentially be attributed to differences in expression and localization of INPP4B isoforms in different tissues, and it would not be surprising if selective misexpression or downregulation of certain isoforms played a role in cancer progression.

Lastly, INPP4B did not appear to be deleted or mutated in human thyroid cancer, but rather, downregulated by gene methylation [5]. The use of epigenetic modifiers to upregulate INPP4B expression or therapeutic targeting of AKT2 may therefore present an effective strategy for the treatment of FTC. However, given the oncogenic role of INPP4B in certain cancers, these novel therapeutic treatments would need to be tailored to specific cancer types and subtypes, a key concept in developing precision medicine for cancer. Furthermore, our findings also suggest that activation of signal transduction pathways associated with endocytic trafficking is critical for tumor cell migration. As a consequence, selective targeting endocytic trafficking and signaling could potentially allow for the development of novel cancer therapies to prevent metastasis.
